# Pediatric Wide-Awake Local Anesthesia No-Tourniquet Hand Surgery: A Practical Approach

**DOI:** 10.1016/j.jhsg.2022.05.002

**Published:** 2022-06-09

**Authors:** Kailash Kapadia, Sejal Shah, Michael Gabriel Galvez

**Affiliations:** ∗ Department of Surgery, Plastic and Reconstructive Surgery Division, Rutgers New Jersey Medical School, New Brunswick, NJ; † Pediatric Hand and Upper Extremity, Pediatric Plastic Surgery, Valley Children’s Healthcare, Madera, CA

**Keywords:** Congenital hand differences, Pediatric hand fractures, Pediatric hand surgery, WALANT, Wide awake

## Abstract

Pediatric wide-awake local anesthesia no-tourniquet technique (WALANT) hand surgery is feasible for the treatment of the spectrum of pediatric hand surgeries, which can include traumatic injuries and congenital hand differences. The key component for success is identifying the appropriate patient for this technique, with the typical patient frequently being >10 years of age. The discussion with the patient and adequate preparation for maximum efficiency become key for patient and surgeon comfort. Here we describe a practical approach for successfully performing this valuable technique for the pediatric population.

Pediatric hand injuries are common and can require operative intervention.^1^, ^2^, ^3^ General anesthesia is frequently required for hand and upper-extremity surgery; however, having the patient completely awake during the procedure can provide several benefits. Wide-awake local anesthesia no-tourniquet technique (WALANT) hand surgery has been established as a reasonable means in the adult population for surgeries that include trigger finger release, open carpal tunnel release, tendon surgery, and more complex reconstruction.^4^ In this technique, no additional medications are administered besides local anesthesia. No tourniquet is used, given that prolonged use results in extremity discomfort and pain.

There are few reports and case series of WALANT hand surgery in children within the literature.^5^^,^^6^ This technique has also been described in Dr Lalonde’s^7^ textbook with his personal approach to the pediatric patient. General anesthesia for children requiring procedures is accepted, particularly with a lack of evidence that it causes adverse neurodevelopmental effects.^8^ Children with complex medical problems, including heart conditions, where general anesthesia could result in a higher risk, may benefit from a WALANT approach. Simple procedures for infants, including postaxial polydactyly in infants, has been well described.**^9^** Finally, there are pediatric patients with complex problems that may require active participation for an intraoperative diagnosis, tendon tensioning after tendon repair or transfers, or digit motion after tenolysis.

Pediatric WALANT hand surgery can be combined with distraction techniques. Distraction in the form of video content can be powerful in children and teenagers.^5^ The readily available smartphone and tablet are frequently used by child life specialists to distract the pediatric population. Providing this unique option to our patients and their families can allow them to feel empowered.^4^ Here, we aim to provide the practical aspects of performing WALANT hand surgery in the pediatric population, with a special focus on children that can participate in the discussion.

## Preoperative Examination

Every patient encounter begins with a history and physical examination. In addition to the traditional history and hand examination, it becomes crucial to observe and assess the patient’s personality and maturity from the moment you enter the room to decide whether they would be an appropriate candidate for the WALANT technique. How does the patient appear during the visit? Do they appear comfortable? Do they appear scared or anxious? Are they engaging in the conversation? Do they appear interested in the discussion? Are they mature for their age? Do they have a history of anxiety? After assessment of the patient, it becomes important to engage in discussion with the parent(s) or guardian(s) and ask similar questions to determine their comfort level. If the patient and guardian appear comfortable and confident, then the patient may be a reasonable candidate for the WALANT approach. Below are lists of indications and contraindications we have observed in our practice that can serve as an initial guide.

## Indications and Contraindications

### Indications

• A compliant patient, typically aged ≥10 years(no absolute age limit)
• Patient actively participating in conversation
• Comfortable and confident patient and guardian
• Congenital hand difference in an older child
• Finger fracture surgery
• Flexor tendon surgery
• Complex tendon surgery

### Contraindications

• A fidgety or restless patient
• Anxious patient or guardian
• History of “not doing well” with needles or vaccine administration
• Any indication of uncertainty from the patient or guardian
• Autistic spectrum (although should be assessed in detail)
• Psychiatric diagnoses (although should be assessed in detail)
• Prolonged operation (>3 hours)
• Surgeon comfort
• “Spidey sense”: unease from the surgeon that a WALANT approach is not indicated

## Preoperative Discussion

The preoperative discussion then becomes the most important aspect for using the WALANT approach, with an emphasis on having clear communication with both the patient and parent. You begin similarly to all other patients, with a description of the etiology of their underlying diagnosis, followed by the proposed surgical intervention. For example, you might describe the fracture, including an x-ray demonstration, then illustrate what has occurred and describe the potential need for operative intervention, which for a fracture can include a closed or open approach followed by fixation, which includes either Kirschner wires or screw and plate placement. Specifically for WALANT patients, it is important for the patient to comprehend the injury and surgery. Therefore, you may have to use adjunctive methods, such as free hand sketches, online images, simplified terminology, and so forth, during your discussion. You then transition to the discussion of anesthesia approaches for the surgery.

At this point, you should already have assessed whether the patient is a good candidate for WALANT hand surgery, which will determine how you proceed with the discussion. Framing the discussion about anesthesia type becomes important: for example, beginning the discussion with general anesthesia, where the patient will be fully asleep, may be agreed upon quickly. Instead, I recommend describing that there are 2 options for the patient, “one is a local anesthesia–only approach where we numb up part of the hand, you won’t feel anything during the surgery, and you will not be watching the surgery” and “the second option is using general anesthesia, where you're fully asleep and then the surgery is performed.” I then look directly at the child and say “numbing you up, so you don’t feel anything and are awake, is something I think we can do for this surgery. During the surgery you will not be watching the surgery but will have the option to: (1) participate, and I will explain the steps as we go; (2) watch a show or movie; or (3) listen to music.” This allows the patient autonomy to choose how they would prefer for us to interface with them during the surgery.

In our experience, most patients select to watch something, so I continue by asking, “what is your favorite movie on Netflix? Or do you prefer Disney Plus?” They then say “Avengers.” Okay, great, which movie exactly? “Avengers: Endgame.” I then respond excitedly, “awesome, well great, you will get to watch that movie during the operation!” I then go into the specifics of what the surgery entails.

The goal is to be transparent about the process to ensure buy-in from the patient and the parent. I then describe that the patient would be taken to the operating room or procedure room, at which point I would start the surgery with injecting the hand with numbing medication. They will hopefully feel just 1 single pinch during the initial needle placement for the anesthetic. I then clarify again that the patient will not be watching the surgery and there will be a hand drape barrier. I always discuss that I will numb up only the portion of the hand that requires surgery. I make eye contact and say that I will only be performing the surgery once they are completely numb. “You won’t feel a thing during the surgery.” If I am unable to achieve sufficient local anesthesia or the patient becomes uncomfortable, then the surgery will be aborted and surgery under general anesthesia will be rescheduled for another day. It is not typically reasonable to have our anesthesia colleagues available to be “on call” for these cases. After the surgery, the patient will be fully awake and will then be able to go to the recovery room awake. After the surgery, the part of the hand that was operated on will be completely numb.

To consider this approach, the ultimate decision and consent must come from the pediatric patient themselves prior to proceeding with WALANT hand surgery. At the end of the visit, you can also ask the patient and parent “what questions do you have?” This open-ended question allows the communication to be complete.

## Surgical Anatomy

The WALANT technique remains the same in children as in adults, except that a lower local anesthetic volume may be needed in a pediatric patient.

## Local Anesthetic

The most common local anesthetics that are used are lidocaine and bupivacaine, with or without epinephrine. It is important to keep in mind the maximum weight-based dosage in the pediatric population. The maximum doses are 5 mg/kg for lidocaine and 2 mg/kg for bupivacaine, and are slightly higher when used with epinephrine.^10^ We typically use a 1:1 mix of 1% lidocaine with 1:100,000 epinephrine and 0.5% bupivacaine and dilute as needed if exceeding the weight-based dose. Bupivacaine is added because it is longer acting. The traditional WALANT approach includes only lidocaine and undiluted 1:100,000 epinephrine. We recommend adding sodium bicarbonate for further patient comfort (1 ml, mixed with 9 ml of local anesthetic for a total of 10 ml).

## Technique

### Streaming show preparation

**Figure 1:**
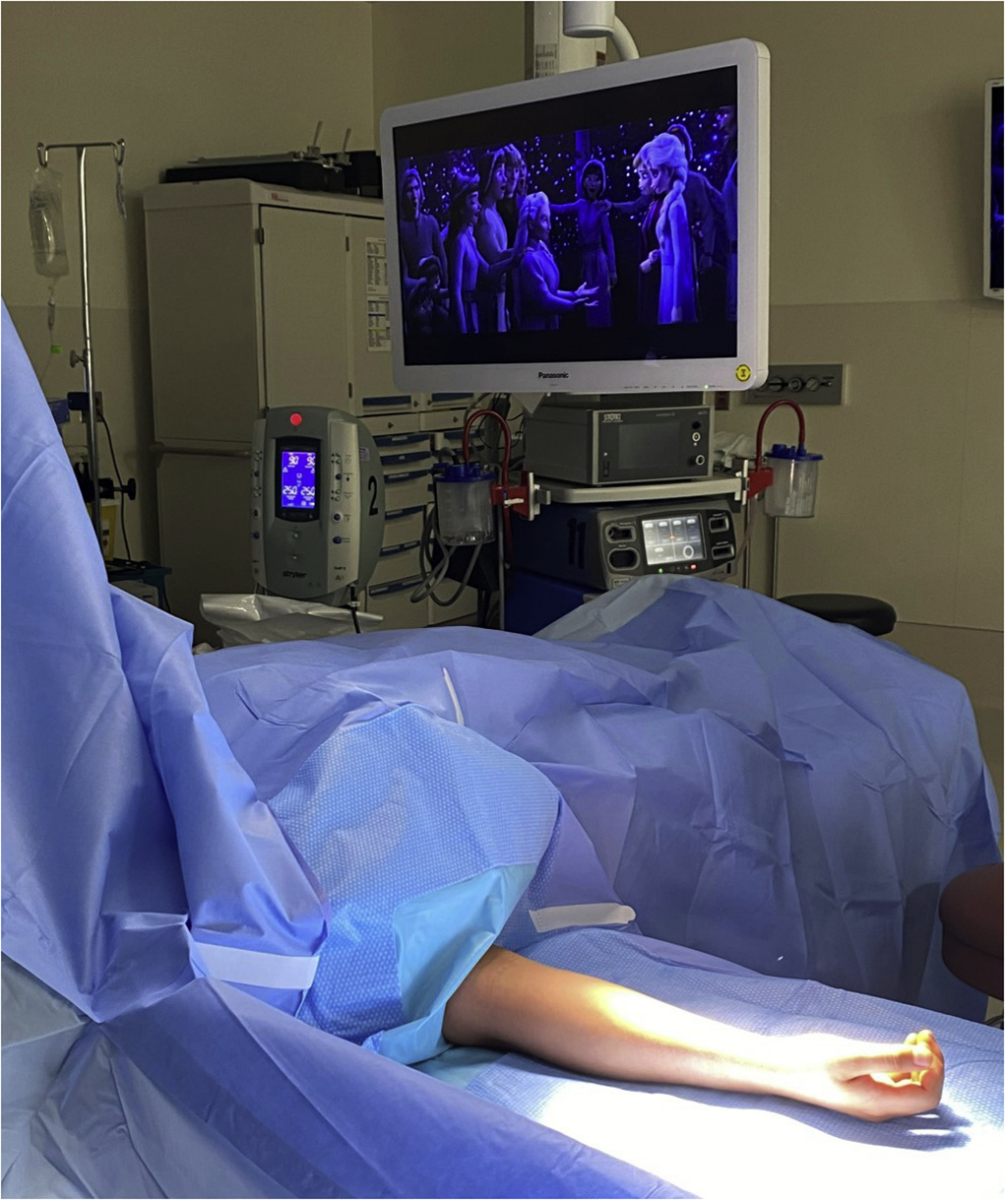
This figure demonstrates a pediatric patient’s upper extremity sterilely draped with a movie playing for distraction.
• Make sure that the show they choose is appropriate for their age and also does not have inappropriate content (check the maturity rating).
• Before the patient enters the operation or procedure room, the show episode or movie is loaded up on the appropriate application.
• The show and volume should only be performed after the time-out.

• The patient is offered “the opportunity” to watch their favorite streaming show while they undergo the procedure (Fig. 1).

### Options for watching the show or episode

**Figure 2:**
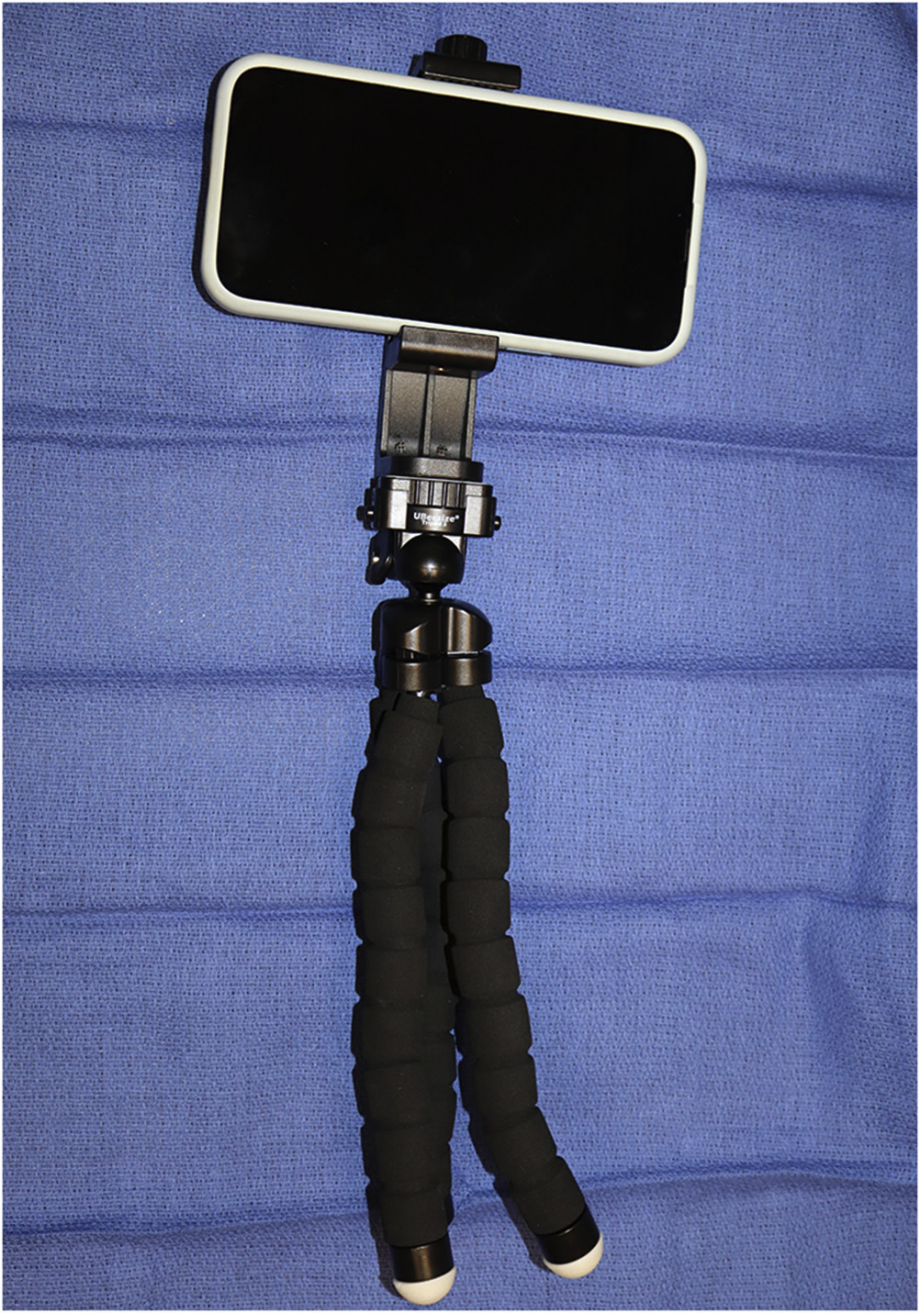
Universal, flexible tripod stands are used to hold a smartphone. These allow the patient to watch a show during WALANT hand surgery.

**Figure 3:**
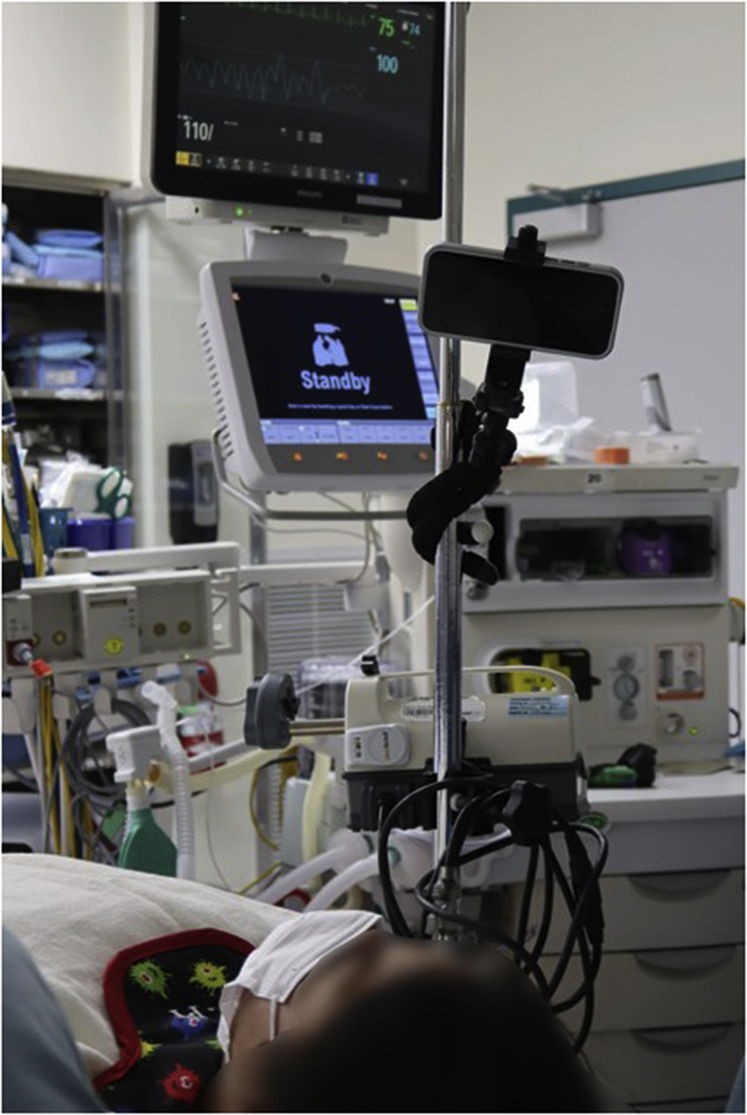
Universal, flexible tripod stands can be easily wrapped around an intravenous pole. Always ask the patient whether the viewing angle is okay for them.
• A tablet (the patient is unable to hold this, and a stand must be provided).
• Overhead operating room screens connected to a computer can be used. Ensure that the streaming website desired is not blocked by your institution.
• A virtual reality platform (that does not require movement of the extremities).
• A tripod stand is required. Having a human hold the phone during the entire operation is not reasonable.
• Speakers with Bluetooth can be used for a “theater effect.”

• A smartphone (larger-screen model preferred) is placed on a flexible tripod stand (Fig. 2) that can be attached to an overhead lamp or intravenous pole (Fig. 3). This should not be placed directly over the patient’s head. If you decide to use your personal smartphone to stream the show, then it is best to put the smartphone on “do not disturb” to avoid text messages being delivered and interrupting the show.

## Immediately Before Surgery

• Make sure to notify the preoperative area not to place an intravenous catheter in the patient.
• Consider keeping the patient nothing by mouth if possible and reasonable; typically I let the patient have something to drink up to 2 hours before the procedure. Although aspiration is unlikely, it is safer to keep them with nothing in their stomach when lying flat for a prolonged period of time during the procedure.
• Do not be afraid to abort the procedure if there is any indication that the patient is anxious (prior to the surgery). I do not recommend any anxiolytics for this operation. The distraction with video means should be sufficient. If the patient has been numbed up safely and the patient has been compliant and distracted, then typically it is safe to proceed with the surgery.
• Prepare the local anesthesia and prefill the syringes before the surgery and before the patient enters the operating or procedure room. Avoid showing the patient the needle or syringes if possible.
• This is a team-based approach with nursing and surgical staff. It is important to keep the patient comfortable during the surgery. You should make sure they are well positioned on the table, with a belt or lead as needed to protect the patient from fluoroscopy.
• Standard monitors are placed as a precaution.
• Always have a sign outside the procedure or operating room door that states “Wide-Awake Surgery.”

## During Surgery

• Always perform the time-out and warn the patient that “we will be talking about you for a moment.” Then, quickly engage the patient after and, at this point, begin the show or movie for distraction.
• Use the drape to block the patient from seeing the injection and procedure.
• Always tell the truth that the initial pinch from the needle will hurt: however, the hope is that this is felt a single time. Have an assistant hold the hand during the injection for safety and to ensure that you only place a single needlestick if possible when performing the local injection.
• “Blow slow as you go.” Take your time. Follow standard WALANT procedures. Have the child take a deep breath in and out when the local anesthetic is initially going in.
• Let the local anesthetic set in before starting the surgery (15–20 minutes). The goal is to avoid having to inject again.
• Always check to ensure that the operative area is adequately numb with an Adson forceps, and ask the patient whether they feel that area being tested.
• While performing the surgery, make sure to engage the patient as you perform the surgery so they are aware that you are going to be manipulating their hand, especially where they may still be sensate.
• Avoid the use of sharp or pain trigger words, such as “needle,” “knife,” “scalpel,” or “scissors.”
• Keep the overall volume of the room down (chatter): this allows the patient to focus on the show and stay fully distracted.
• Ask the patient whether they would like to see an aspect of the operation, if this includes movement or what the wound looks like. Many patients may want to see what it looks like before the dressing is placed.
• Having the patient see the wound will allow them to “respect” the work performed and further protect the wound during healing.
• Remember during casting and dressing application that the patient can hold up their arm if you ask them to.
• At the end of the operation, I ask them whether they would do it this way again. I also ask them what our “Yelp” review would be out of 5 stars. You will be surprised that they are satisfied with this approach.

## Postoperative Management

The child is also educated on how to take care of their hand during the procedure. This will, for example, involve instructions suggesting the importance of the orthosis and dressing, the weight-bearing status of their hand, ranges of movements that are safe, instructions regarding pain medication, and/or future plans for hand therapy or recovery. They also have no pain after the surgery and can be easily discharged home after the surgery. The patient can bypass the Postanesthesia Care Unit and be transferred straight to the recovery room, which may be another advantage to this technique for the patient and the family.

## Perils and Pitfalls or Complications

We have not had any complications from the use of local anesthesia in place of general anesthesia. We discussed many of the perils and pitfalls, from difficulty with preoperative counseling to possibly having to abort the procedure before the start of the case. And we hope this manuscript provides you with the tools to consider WALANT for your pediatric patients.

## Case Illustrations

### Case 1

**Figure 4:**
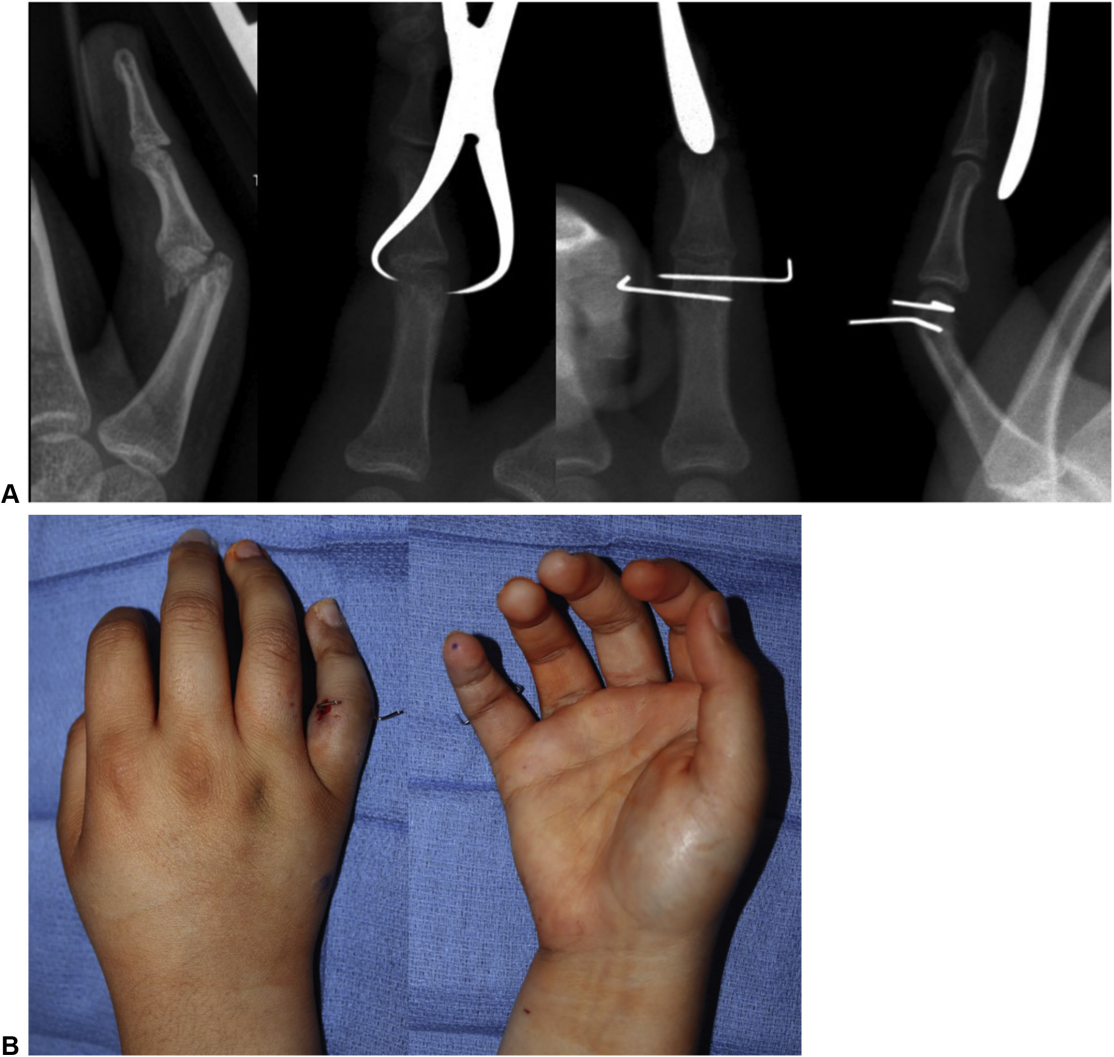
Case 1: WALANT little finger proximal phalanx unicondylar closed reduction and percutaneous pinning. This patient underwent WALANT closed reduction with a towel clamp, followed by transverse pinning with Kirshner wires across the fracture.

A 13-year-old girl sustained a right, little finger, proximal phalanx, unicondylar fracture while playing softball. She underwent WALANT hand surgery with closed reduction and percutaneous pinning (Fig. 4). Her fracture healed and she regained normal motion of the finger. This patient watched a Disney movie during her surgery. This patient, when asked about the wide-awake approach, stated that she would do it again under local anesthesia.

### Case 2

**Figure 5:**
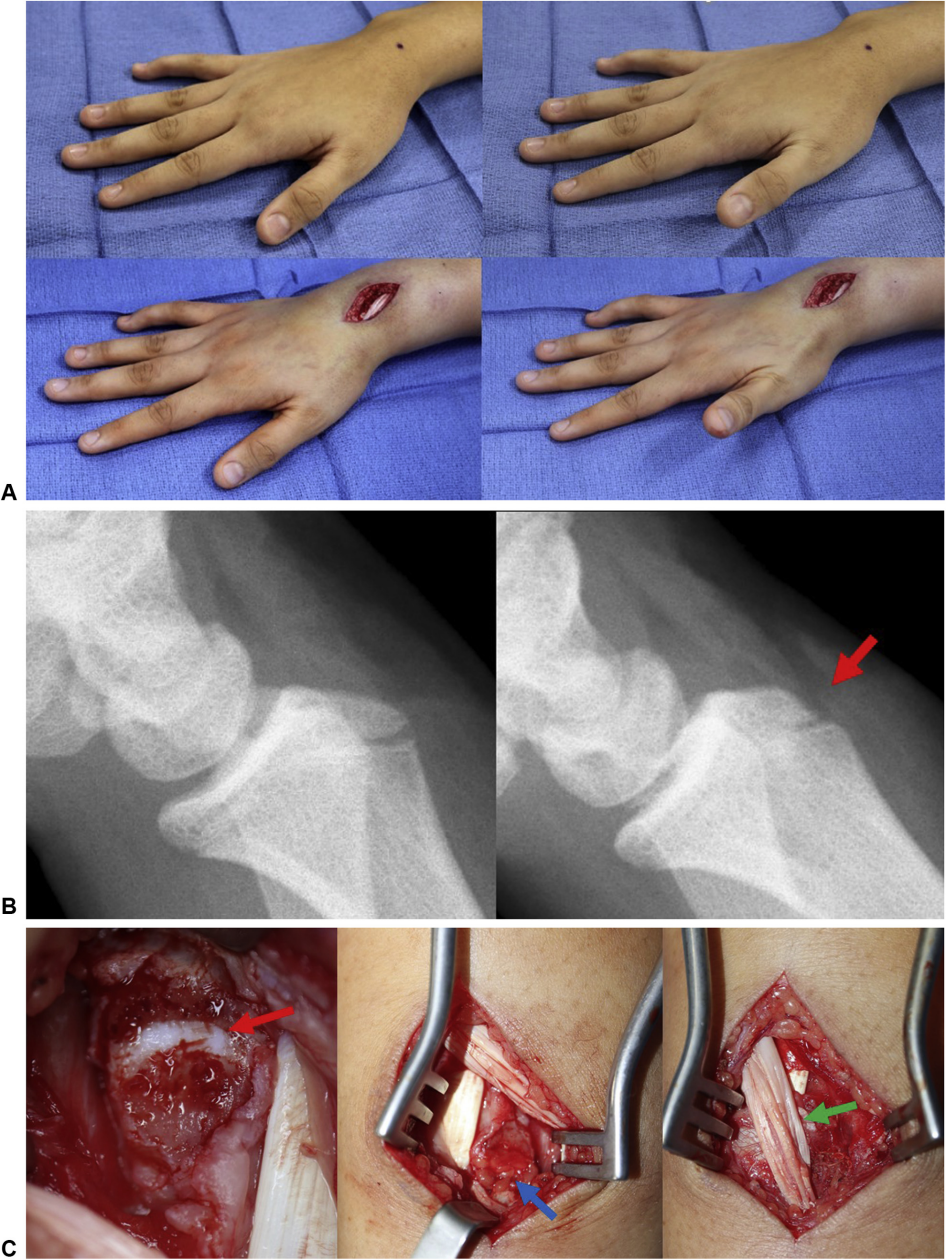
Case 2: extensor pollicis longus WALANT exploration. **A** This patient underwent WALANT hand surgery with tenolysis and findings of a sharp Lister tubercle. Before surgery, she was unable to retropulse (photos above); after tendon release, she was able to retropulse normally and without pain (photos below). **B** An osteotome was used to excise the prominent tubercle (red arrow). **C** The physis of the distal radius was open (red arrow); therefore, a local fat graft was placed over this physis (blue arrow). The frayed extensor pollicis longus tendon was externalized (green arrow).

A 14-year-old girl had a 1-year history of right wrist pain after a fall. Her examination was significant with pain within the third extensor compartment and incomplete retropulsion. Magnetic resonance imaging demonstrated tenosynovitis of the extensor pollicis longus. She underwent WALANT hand surgery with tenolysis and findings of a sharp Lister tubercle (Fig. 5). She underwent excision of the Lister tubercle, with local fat grafting over the open physis, and the frayed extensor pollicis longus tendon was externalized. She regained normal thumb retropulsion and was pain free after surgery. This patient watched “Grey’s Anatomy” during her surgery. This patient, when asked about the wide-awake approach, stated that she would do it again under local anesthesia.
